# Chromosome-level genome assembly of the critically endangered Baer’s pochard (*Aythya baeri*)

**DOI:** 10.1038/s41597-023-02063-9

**Published:** 2023-03-29

**Authors:** Lei Zhang, Xiaodong Gao, Tian Xia, Xiufeng Yang, Guolei Sun, Chao Zhao, Guangshuai Liu, Honghai Zhang

**Affiliations:** College of Life Sciences, Qufu Normal University, Qufu, 273165 Shandong China

**Keywords:** Evolutionary ecology, Molecular evolution

## Abstract

Baer’s pochard (*Aythya baeri*) is a critically endangered species historically widespread throughout East Asia, whose population according to a recent estimate has decreased to between 150 and 700 individuals, and faces a long-term risk of extinction. However, the lack of a reference genome limits the study of conservation management and molecular biology of this species. We therefore report the first high-quality genome assembly of Baer’s pochard. The genome has a total length of 1.14 Gb with a scaffold N50 of 85,749,954 bp and a contig N50 of 29,098,202 bp. We anchored 97.88% of the scaffold sequences onto 35 chromosomes based on the Hi-C data. BUSCO assessment indicated that 97.00% of the highly conserved Aves genes were completely present in the genome assembly. Furthermore, a total of 157.06 Mb of repetitive sequences were identified and 18,581 protein-coding genes were predicted in the genome, of which 99.00% were functionally annotated. This genome will be useful for understanding Baer’s pochard genetic diversity and facilitate the conservation planning of this species.

## Background & Summary

**Fig. 1:**
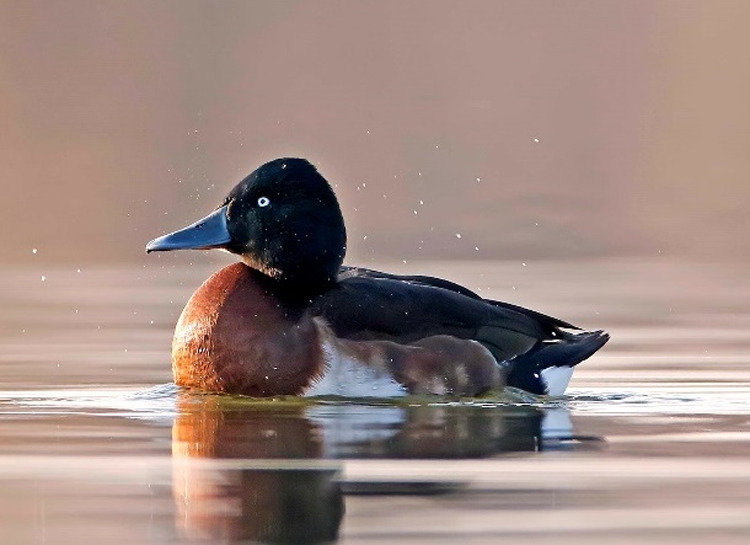
An adult male Baer’s pochard.(Qiang Li).

Baer’s pochard is a migratory duck belonging to the order Anseriformes, family Anatidae, and genus *Aythya*, whose closest relative and sister species is the ferruginous duck^1^. Baer’s pochard has typical sexual dimorphism. Males have white or light-yellow irises (Fig. 1), whereas females have dark brown irises. Females also have reddish brown spots at the base of the beak^2,3^, and are smaller in size. This species was once widespread in East and South Asia, but is currently predominantly only in China^4,5^ due to over-exploitation and habitat loss, which have caused a severe and global population decline over the past decades^6,7^. Baer’s pochard was classified as endangered by the International Union for Conservation of Nature (IUCN) in 2008, then as Critically Endangered in 2012, and in 2021 was included in the China Red Data Book of Endangered Animals. According to a recent estimate by the IUCN, its population has only 150–700 mature individuals^8^, and faces a long-term risk of extinction. Moreover, although there has been an increasing number of avian genome assemblies in recent years^9^, many non-model species including Baer’s pochard still lack genome resources.

In order to provide genome-scale insights into a near-extinction species and promote conservation planning for it, we constructed the first high-quality Baer’s pochard chromosome-level reference genome using Illumina paired-end sequencing, Oxford Nanopore sequencing, and Hi-C technology. The genome had an assembly size of 1.14 Gb with a scaffold N50 of 85,749,954 bp and a contig N50 of 29,098,202 bp. These scaffolds were further clustered and ordered into 35 pseudo-chromosomes based on the Hi-C data, representing 97.88% of the assembled sequences. The genome contained 13.72% repeat sequences and 1,721 noncoding RNAs. A total of 18,581 protein-coding genes were predicted in the genome, of which 99.00% were functionally annotated. Searches for complete Aves BUSCO (Benchmarking Universal Single-Copy Ortholog) gene groups showed that 97.00% of BUSCO genes were complete, suggesting a high level of genome completeness. This genome provides a valuable genomics resource for studying the conservation genomics of critically endangered species to help recover their population size.

## Methods

### Ethics statement

All animal handling and experimental procedures were approved by the Qufu Normal University Biomedical Ethics Committee (approval number: 2022001).

### Sample and sequencing

Baer’s pochard tissue for whole-genome sequencing was obtained from a dead individual that had strayed into a fishing net in Shandong (China). The muscle tissue that we collected was stored at −80 °C and used for genomic DNA extraction, genomic DNA sequencing. Nine additional transcriptomic samples (heart, kidney, lung, spleen, liver, craw, gallbladder, blood, and muscle) were collected from the same individual and stored at −80 °C until RNA were extracted for transcriptome sequencing. Paired-end libraries of genomic DNA (gDNA) were prepared using Illumina TruSeq Nano DNA Library Prep kits. The integrity and quality of the extracted DNA were checked using agarose gel electrophoresis and a Qubit Fluorometer. One library with an insertion size of 350 bp was constructed and sequenced using the Illumina HiSeq platform to enable genome survey and base-level correction. A total of 60.34 Gb (coverage of 49.69×) of 150-bp paired-end reads were generated. Purified DNA was then prepared for sequencing with the genomic sequencing kit SQK-LSK109 (Oxford Nanopore Technologies, Oxford, UK) following the provided protocol, and single-molecule real-time sequencing of long reads was conducted using the PromethION platform (ONT, Oxford, UK). Approximately 136.50 Gb of data was obtained (coverage of 112.42×). The Hi-C library was constructed using muscle tissue from the same Baer’s pochard individual and sequenced using the Illumina PE150 platform. A total of 125.64 Gb of 150-bp paired-end reads were obtained, which covered ~103.48× of the genome (Table 1). Finally, RNA was extracted from the nine transcriptomic samples and used for library construction, and RNA-Seq reads were generated for genome annotation using the Illumina NovaSeq 6000 platform. A total of 67.93 Gb of 150-bp paired-end reads were obtained after adapter trimming and quality filtering (Table 2).

**Table 1:** Sequencing data for *A. baeri* genome assembly.

Sequencing Strategy	Sequencing platform	Library size (bp)	Total data (Gb)	Sequence coverage (X)
Illumina	Illumina HiSeq	350	60.34	49.69
Nanopore	PromethION	20 kb	136.50	112.42
Hi-C	Illumina PE150	350	125.64	103.48
Total	—	—	322.48	265.59

**Table 2:** Statistical analysis of transcriptome sequencing results of nine organs.

Sample	Raw Reads	Clean Reads	Raw Base (Gb)	Clean Base (Gb)	Q20(%)	Q30(%)	GC Content (%)
blood	########	20,670,905	6.25	6.20	97.57	93.44	53.83
crop	########	23,991,244	7.25	7.20	97.74	93.93	51.83
liver	########	23,520,077	7.17	7.06	98.09	94.68	51.05
spleen	########	27,138,546	8.19	8.14	98.00	94.48	56.16
muscle	########	24,792,681	7.84	7.44	97.69	93.66	52.43
kidney	########	26,158,054	7.91	7.85	97.71	93.87	51.43
gallbladder	########	27,915,285	8.44	8.37	97.55	93.48	53.39
lung	########	25,520,350	7.71	7.66	97.81	94.06	51.25
heart	########	26,692,737	8.08	8.01	97.35	92.88	51.55

### Genome assembly

**Fig. 2:**
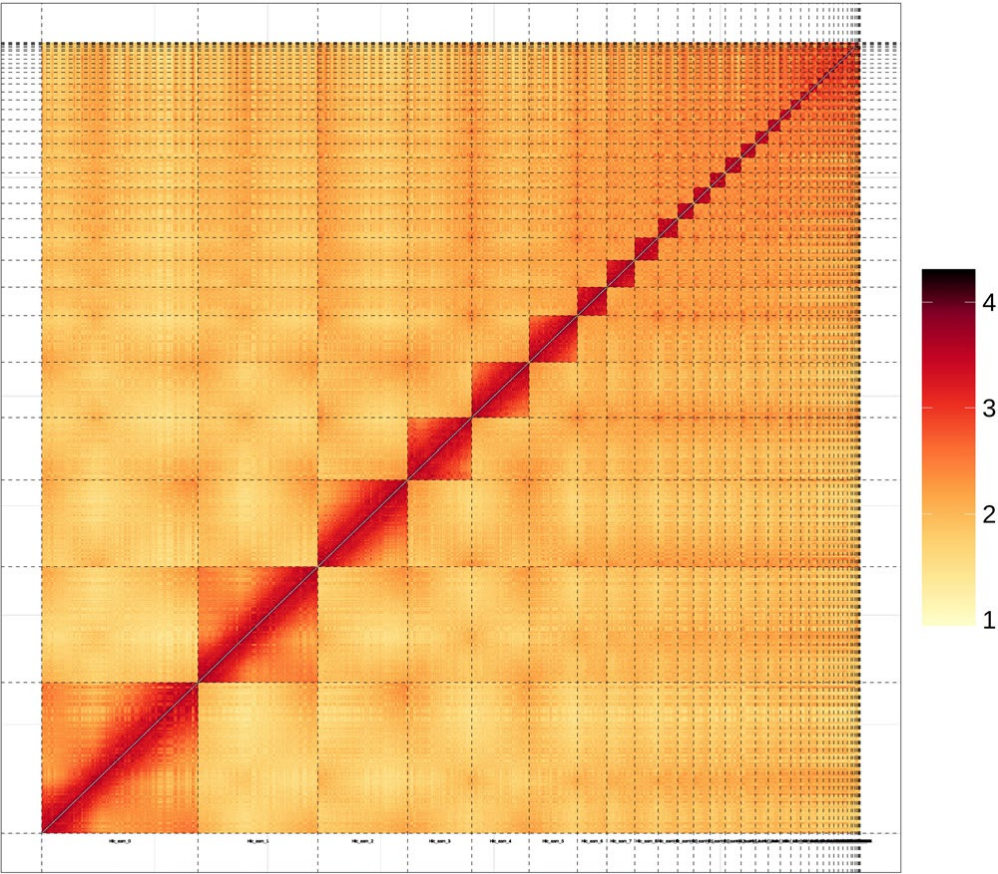
Heat map of Hi-C assembly of the Baer’s pochard.

We used a combination of Nanopore long reads, Illumina short reads, and chromatin conformation capture (Hi-C) to generate chromosome-level reference genomes. The genome size and heterozygosity level of the Baer’s pochard were determined using Illumina short reads based on the k-mers spectrum^10^. The genome size was estimated to be approximately 1,214.25 Mb, and the heterozygosity rate of the genome is 0.38% (Table 3). NextDenovo (https://github.com/Nextomics) used Nanopore long reads for the initial scaffolding assemblies. However, long reads have low quality scores, and thus NextPolish^11^— which uses quality-controlled Illumina short reads, was employed to improve the assembled genome. These steps yielded the final Baer’s pochard genome with a total length of 1.14 Gb, which was mostly consistent with the k-mer-based estimation including 228 contigs with N50 = 29,098,202 bp, and the overall GC content of the genome was 41.94% (Table 4). We had obtained 125.64 Gb of Hi-C sequencing data to generate this chromosomal-level assembled genome. We first used HiCUP^12^ to map and process the reads obtained from the Hi-C library, then the Hi-C-corrected contigs were subjected to the ALLHiC pipeline^13^ for partition, orientation and ordering. A total of 135 scaffolds could be mapped to 35 chromosomes with lengths ranging from 1.77 Mb to 208.01 Mb, which covered 97.88% of the whole genome. Finally, we obtained the first chromosome-level high-quality Baer’s pochard assembly (1.14 Gb) with a scaffold N50 length of 85.75 Mb (Table 5 and Fig. 2). The genome size, scaffold N50 length, and GC content of *Aythya baeri* is similar to that of *Aythya fuligula* (RefSeq assembly access: GCF_009819795.1), a member of the same genus, but its contigN50 length is much longer than that of *Aythya fuligula* (Table 6). This indicates that the genome of *Aythya baeri* has high assembly quality.

**Table 3:** K-mer frequency and genome size evaluation of *A. baeri*.

Kmer	K-mer Depth	K-mer number	Genome size (Mb)	Revised Genome size (Mb)	Heterozygous rate (%)	Repeat rate (%)
17	34	41,976,983,695	1,234.62	1,214.25	0.38	38.82

**Table 4:** The result of A. baeri genome assembly.

Title	Total length	Total number	Average length	Max length	Min length	N50 length	N50 number	N90 length	N90 number
Contig	###########	228	5,020,981	########	19,035	########	12	6,338,495	44

**Table 5:** Chromosome and reference genome corresponding chromosome statistical results.

Chr ID	Cluster Number	Size (bp)
Chr 1	6	208,009,351
Chr 2	4	160,030,598
Chr 3	9	120,378,128
Chr 4	4	77,276,889
Chr 5	3	65,105,551
Chr 6	3	40,107,664
Chr 7	2	37,709,550
Chr 8	2	32,466,243
Chr 9	2	26,841,654
Chr 10	3	22,316,684
Chr 11	3	22,074,026
Chr 12	2	21,639,348
Chr 13	2	21,513,030
Chr 14	3	20,405,955
Chr 15	2	18,111,443
Chr 16	4	16,439,097
Chr 17	2	15,421,515
Chr 18	3	13,744,401
Chr 19	4	12,227,774
Chr 20	3	12,226,005
Chr 21	6	8,780,536
Chr 22	2	8,665,845
Chr 23	2	7,751,464
Chr 24	4	6,913,257
Chr 25	2	6,873,023
Chr 26	4	6,552,211
Chr 27	4	6,165,718
Chr 28	2	3,330,631
Chr 29	2	3,274,723
Chr 30	14	3,385,386
Chr 31	4	2,644,619
Chr 32	3	2,416,163
Chr 33	2	2,192,944
Chr 34	2	1,771,048
Chr Z	9	85,749,954

**Table 6:** Comparative analysis of the genome of *A. baeri* and *A. fuligula*.

Species	Genome size (Gb)	Number of scaffolds	Scaffold N50 (Mb)	Scaffold L50	Number of contigs	Contig N50 (Mb)	Contig L50	GC percent (%)
*A. baeri*	1.14	135	85.75	4	228	29.10	12	41.94
*A. fuligula*	1.13	104	85.91	4	267	17.81	19	41.50

We used the Core Eukaryotic Genes Mapping Approach (CEGMA v2.5)^14^ and Benchmarking Universal Single-Copy Orthologs (BUSCO v4.1.2)^15^ methods to evaluate the completeness of genome assembly. A single-copy ortholog set was searched against the assembled genome of Baer’s pochard using BUSCO tool, of the 8,338 single-copy orthologs in the avian lineage (aves_odb10), approximately 97.00% were present in this assembly (Table 7). We took the conserved genes (248 genes) of six eukaryotic model organisms to form the core gene library, of which the CEGMA evaluation showed 95.97% was successfully assembled (Table 8).

**Table 7:** BUSCO analysis result of *A. baeri* genome.

Category	Number	Ratio (%)
Complete BUSCOs	8,089	97.00
Complete and single-copy BUSCOs	8,061	96.70
Complete Duplicated BUSCOs	28	0.30
Fragmented BUSCOs	87	1.00
Missing BUSCOs	162	2.00
Total BUSCO groups searched	8,338	—

**Table 8:** Statistical evaluation of genomic integrity by CEGMA.

Species	complete		complete + partial	
	Prots	Completeness (%)	Prots	Completeness (%)
*A. baeri*	234	94.35	238	95.97

### Annotation of genomic repeat sequences

We annotated the Baer’s pochard whole-genome repeat sequences based on homology alignment and de novo predictions. RepeatModeler (v1.0.8)^16^, RepeatScout (v1.0.5)^17^ and LTR_FINDER (v1.0.7)^18^ were used to build a de novo repetitive element database. Tandem repeats were extracted using TRF^19^ via ab initio prediction. Homolog prediction was performed using the Repbase database^20^ whilst employing the RepeatMasker (v4.0.5) software^21^ to extract repeat regions (Table 9). According to these analyses, approximately 1,571 Mb of repeat sequences were revealed, which accounted for 13.72% of the whole genome; thus, the content of repeat sequence in *A. baeri* genome is slightly higher than that in the *A. fuligul* genome (13.00%). Among the repeat elements, long interspersed nuclear elements (LINEs) account for 8.80% of the genome, short interspersed nuclear elements (SINEs) for 0.01%, long terminal repeats (LTRs) for 4.13% and DNA transposons for 0.15% (Table 10).

**Table 9:** Annotation of repeated sequences.

Type	Repeat Size	% of genome
Trf	32,934,684	2.88
Repeatmasker	136,239,078	11.90
Proteinmask	58,496,877	5.11
Total	157,096,819	13.72

**Table 10:** Repetitive elements and their proportions in *A. baeri* genome.

Type	Denovo + Repbase		TE Proteins		Combined TEs	
	Length (bp)	Percentage (%)	Length (bp)	Percentage (%)	Length (bp)	Percentage (%)
DNA	1,516,832	0.13	230,564	0.02	1,726,412	0.15
LINE	92,362,357	8.07	49,394,908	4.31	100,765,525	8.80
SINE	154,947	0.01	0	0	154,947	0.01
LTR	45,132,101	3.94	8,899,775	0.78	47,262,135	4.13
Unknown	5,157,967	0.45	0	0	5,157,967	0.45
Total	#########	11.90	58,496,877	5.11	139,971,152	12.23

### Annotation of gene structure

We combined three approaches to predict protein-coding genes, including homologous comparison, ab initio prediction, and RNA-Seq-assisted prediction. For homologous comparison, the reference protein sequences of five bird species— the tufted duck (*Aythya fuligula*), mallard (*Anas platyrhynchos*), mute swan (*Cygnus olor*), red junglefowl (*Gallus gallus*), and ruddy duck (*Oxyura jamaicensis*), were sourced from the Ensembl database (release 91), and aligned to the Baer’s pochard genome using TBlastN (v2.2.26; E-value ≤ 1e-5)^22^. The potential gene structures were predicted using Genewise (v2.4.1)^23^. For ab initio analysis based gene prediction, we used Augustus (v3.2.3)^24^, Geneid (v1.4)^25^, Genescan (v1.0)^26^, GlimmerHMM (v3.04)^27^ and SNAP^28^ with appropriate parameters to perform de novo predictions. To optimize the genome annotation, RNA-Seq reads from nine different tissues were assembled de novo using Trinity (v2.1.1)^29^, and TopHat (v2.0.11)^30^ was used to align RNA-seq reads to the Baer’s pochard genome sequences. Cufflink software was then employed to determine potential gene structures. We used EvidenceModeler (EVM,v1.1.1) and PASA (Program to Assemble Spliced Alignment) to integrate all the results generated from the three aforementioned methods and create a non-redundant reference gene set^31^ composed of 18,581 genes, with an average CDS lengths of 1,600.42 bp, average exon and intron lengths were 169.04 bp and 2,763.57 bp, respectively (Table 11).

**Table 11:** Prediction of protein-coding genes.

Methods/Tools		Gene number	Average exons per gene	Average length (bp)			
				transcript	CDS	Exon	Intron
De novo	Augustus	17,152	8.91	18,793.13	1,528.97	171.52	2,181.40
	GlimmerHMM	163,564	3.04	6,181.26	527.60	173.27	2,764.75
	SNAP	62,717	5.72	27,696.05	678.33	118.64	5,727.27
	Geneid	28,481	7.09	26,694.29	1,300.98	183.49	4,169.66
	Genscan	37,873	8.47	22,684.68	1,428.19	168.69	2,846.94
Homolog	*A. fuligula*	18,627	8.56	19,214.87	1,526.82	178.42	2,340.52
	*A. platyrhynchos*	36,236	5.31	10,485.21	1,050.51	197.96	2,190.68
	*C. olor*	28,808	6.09	12,846.43	1,185.84	194.68	2,290.32
	*G. gallus*	26,748	6.14	13,145.65	1,215.00	197.76	2,319.45
	*O. jamaicensis*	26,843	6.31	13,394.28	1,208.86	191.43	2,292.67
RNAseq	PASA	71,974	6.96	17,919.21	1,193.44	171.38	2,804.62
	Cufflinks	61,007	10.06	30,770.70	3,804.03	378.18	2,976.82
EVM		20,267	9.05	23,673.42	1,525.73	168.65	2,752.40
Pasa-update		20,176	8.96	23,602.89	1,527.25	170.39	2,772.27
Final set		18,581	9.47	25,001.31	1,600.42	169.04	2,763.57

We also predicted 432 tRNAs using the program tRNAscan-SE^32^. We identified 664 ncRNAs, including 342 miRNAs and 322 snRNAs, by searching against the Rfam database with default parameters using Infernal^33^. For rRNAs that were highly conserved, we chose related species’ rRNA sequences as references and predicted 161 rRNA sequences using Blast^34^ (Table 12).

**Table 12:** Annotation of non-coding RNA genes.

Type		Copy	Average length (bp)	Total length (bp)	% of genome
miRNA		342	88.48	30,260	0.002643
tRNA		432	75.16	32,467	0.002836
rRNA	rRNA	161	199.45	32,112	0.002805
	18 S	14	477.57	6,686	0.000584
	28 S	59	253.86	14,978	0.001308
	5.8 S	3	156.00	468	0.000041
	5 S	85	117.41	9,980	0.000872
snRNA	snRNA	322	128.93	41,517	0.003627
	CD-box	124	96.75	11,997	0.001048
	HACA-box	82	142.26	11,665	0.001019
	splicing	97	150.93	14,640	0.001279

### Functional annotation of protein-coding genes

**Fig. 3:**
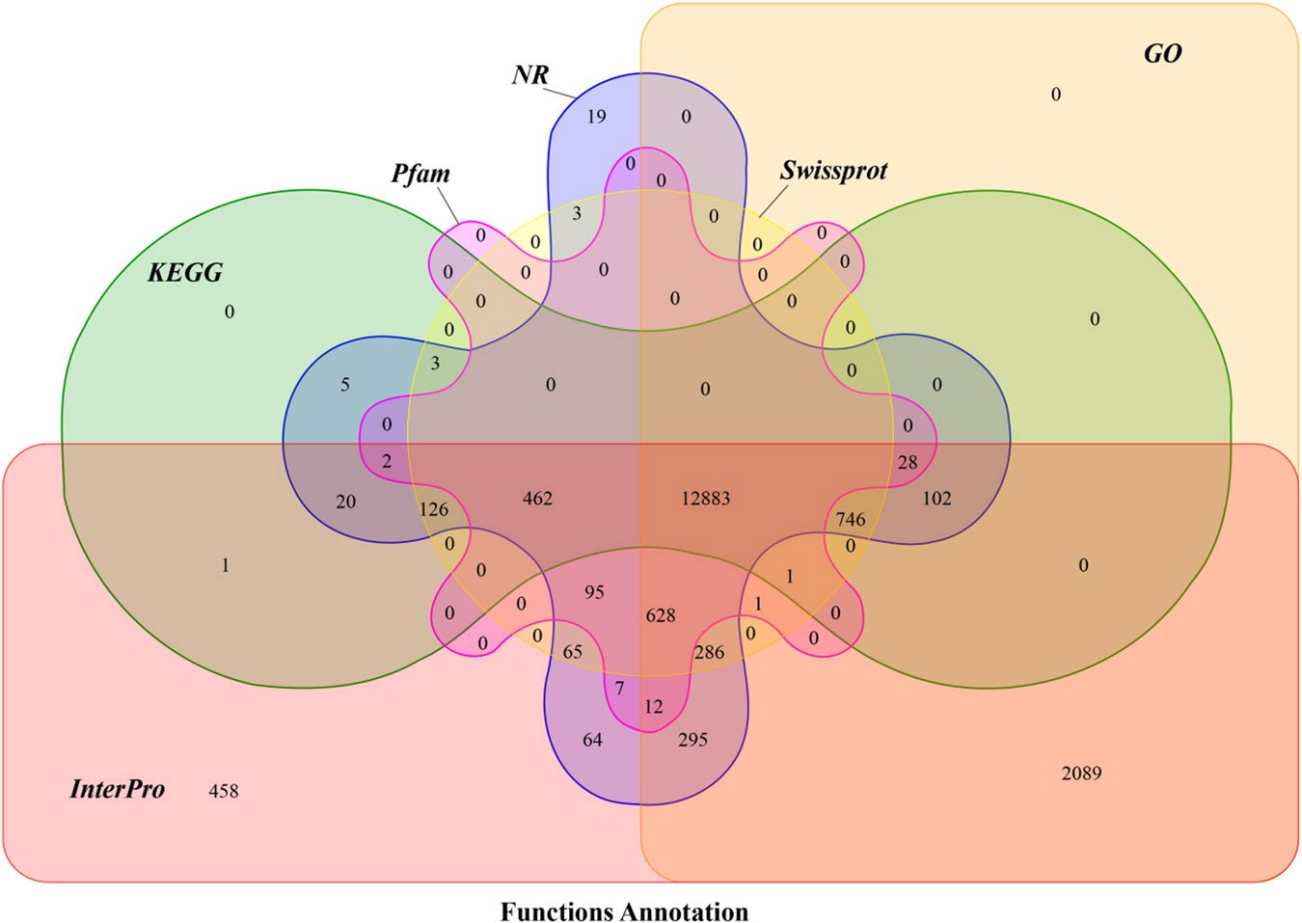
Functional annotation statistics. Venn diagram illustrating the distribution of high-score matches of the functional annotation in the Baer’s pochard genome against six public databases.

We functionally annotated the predicted proteins in the Baer’s pochard genome according to homologous searches against six databases: SwissProt^35^, InterPro^36^, Pfam^37^, Kyoto Encyclopedia of Genes and Genomes (KEGG)^38^, Gene Ontology (GO)^39^, and Nr (http://www.ncbi.nlm.nih.gov/protein). Respectively, 82.39%, 98.90%, 76.00%, 77.40%, 91.90%, and 85.30% of genes matched the database entries (Fig. 3). In summary, 18,401 genes (99.00%) were successfully annotated by gene function and conserved protein motifs (Table 13).

**Table 13:** Functional annotation of the predicted protein-coding genes.

Methods for annotation	Number	Percent (%)
Total	18,581	—
Swissprot	15,299	82.30
Nr	15,851	85.30
KEGG	14,379	77.40
InterPro	18,371	98.90
GO	17,071	91.90
Pfam	14,119	76.00
Annotated	18,401	99.00
Unannotated	180	1.00

### Synteny analysis using the Tufted duck genome

**Fig. 4:**
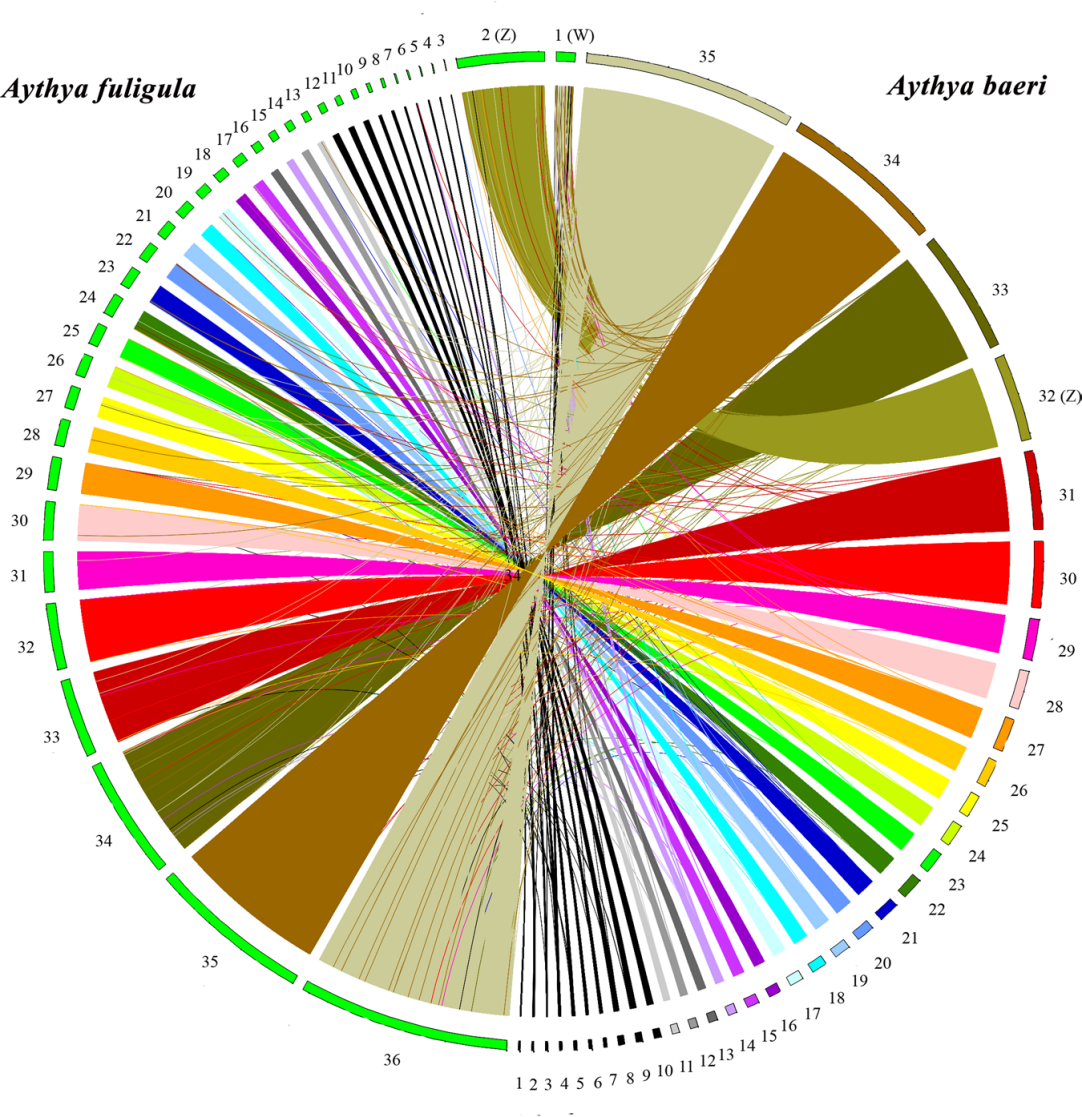
Circos plot of the synteny analysis between the tufted duck and the Baer’s pochard genome.

We conducted whole-genome synteny analysis between the Tufted duck (GCA_009819795.1) and the Baer’s pochard genomes using MUMmer^40^. The whole-genome alignment between the tufted duck and the Baer’s pochard genomes was visualized using RectChr (BGI-shenzhen/RectChr), as shown in Fig. 4. The results showed the overall high consistency of the tufted duck and the Baer’s pochard genomes.

## Data Records

The Nanopore, Illumina, and Hi-C sequencing data used for genome assembly were deposited in the NCBI Sequence Read Archive database with accession numbers SRR17568785^41^, SRR17518553^42^, and SRR17509905^43^. The transcriptomic sequencing data were stored under accession numbers SRR17433182^44^ and SRR17497023^45^-SRR17497030. The assembled genome was deposited in the NCBI assembly with the accession number JAKRSJ000000000^46^. The annotation results of repeated sequences, gene structure and functional prediction were deposited in the Figshare database^47^.

## Technical Validation

The integrity of the extracted DNA was checked by agarose gel electrophoresis, and the main band was found to be approximately 45 Kb long. The concentration of DNA was determined using a Qubit fluorometer (Thermo Fisher Scientific, USA) with an absorbance of approximately 1.80 at 260/280.

We used the sequence identity method to evaluate the completeness of the genome assembly, selected small fragment library reads, and used BWA software (http://bio-bwa.sourceforge.net/) to align them with the assembled genome. The alignment rate of all small fragment reads to the genome was approximately 99.71%, and the coverage rate was approximately 99.45%, indicating consistency between the reads and assembled genome.

SNPs were identified using Samtools (v0.1.19), resulting in the identification of 3,162,696 SNPs, including 3,157,033 heterozygous SNPs and 5,663 homozygous SNPs. The proportion of homozygous SNPs was 0.000502%, indicating the high accuracy of this assembly.

## Data Availability

All commands and pipelines used in data processing were executed according to the manual and protocols of the corresponding bioinformatic software. No specific code has been developed for this study.
